# Translation of a tailored nutrition and resistance exercise intervention for elderly people to a real-life setting: adaptation process and pilot study

**DOI:** 10.1186/s12877-017-0413-8

**Published:** 2017-01-18

**Authors:** Ellen JI. van Dongen, Joanne N. Leerlooijer, Jan M. Steijns, Michael Tieland, Lisette CPGM. de Groot, Annemien Haveman-Nies

**Affiliations:** 1 Division of Human Nutrition, Wageningen University, PO Box 8129, 6700 EV Wageningen, The Netherlands; 2 FrieslandCampina Institute, P.O. Box 1551, 3800 BN Amersfoort, The Netherlands

**Keywords:** Adaptation, Feasibility, Resistance exercise, Protein-rich products, Elderly, Real-life setting

## Abstract

**Background:**

Combining increased dietary protein intake and resistance exercise training for elderly people is a promising strategy to prevent or counteract the loss of muscle mass and decrease the risk of disabilities. Using findings from controlled interventions in a real-life setting requires adaptations to the intervention and working procedures of healthcare professionals (HCPs). The aim of this study is to adapt an efficacious intervention for elderly people to a real-life setting (phase one) and test the feasibility and potential impact of this prototype intervention in practice in a pilot study (phase two).

**Methods:**

The Intervention Mapping approach was used to guide the adaptation in phase one. Qualitative data were collected from the original researchers, target group, and HCPs, and information was used to decide whether and how specified intervention elements needed to be adapted. In phase two, a one-group pre-test post-test pilot study was conducted (*n* = 25 community-dwelling elderly), to elicit further improvements to the prototype intervention. The evaluation included participant questionnaires and measurements at baseline (T0) and follow-up (T1), registration forms, interviews, and focus group discussions (T1). Qualitative data for both phases were analysed using an inductive approach. Outcome measures included physical functioning, strength, body composition, and dietary intake. Change in outcomes was assessed using Wilcoxon signed-rank tests.

**Results:**

The most important adaptations to the original intervention were the design of HCP training and extending the original protein supplementation with a broader nutrition programme aimed at increasing protein intake, facilitated by a dietician. Although the prototype intervention was appreciated by participants and professionals, and perceived applicable for implementation, the pilot study process evaluation resulted in further adaptations, mostly concerning recruitment, training session guidance, and the nutrition programme. Pilot study outcome measures showed significant improvements in muscle strength and functioning, but no change in lean body mass.

**Conclusion:**

The combined nutrition and exercise intervention was successfully adapted to the real-life setting and seems to have included the most important effective intervention elements. After adaptation of the intervention using insights from the pilot study, a larger, controlled trial should be conducted to assess cost-effectiveness.

**Trial registration:**

Trial registration number: ClinicalTrials.gov NL51834.081.14 (April 22, 2015).

## Background

In aging societies, increased attention is being given to strategies to maintain independent functioning among older adults. Sarcopenia, the age-related loss of muscle mass and strength in elderly people [1–3], contributes to loss of physical functioning [3] and subsequently increases challenges to living at home independently in the long term [4]. Meta-analyses showed that the combination of resistance-type exercise training and dietary protein supplementation augments muscle mass and improves strength and physical performance [5, 6] and proved a promising strategy to counteract sarcopenia. In the Netherlands, a randomised double-blind placebo controlled trial investigated the impact of daily protein supplementation during twice-weekly resistance-type exercise training in (pre-)frail older adults. This RCT was performed in an academic setting, facilitating high compliance, and the intervention activities, including the training sessions, were implemented by researchers. All participants performed the resistance exercise twice a week, and participants consumed either a dairy protein drink or a placebo drink after both breakfast and lunch every day. The study’s findings were promising and showed a significant increase in muscle mass and strong improvements in muscle strength and physical performance after 12 and 24 weeks of intervention [7]. Implementing this efficacious intervention in real-life practice may benefit the health of community-dwelling elderly people. However, since efficacy interventions differ from effectiveness interventions, it is not certain that these findings can be directly translated into a real-life setting [8], for instance communities or care organisations. To increase the likelihood of achieving similar effects in a real-life setting, the efficacious intervention should be adapted to fit the practice setting.

To our knowledge, adaptation processes for combined nutrition and exercise interventions for community-dwelling elderly have not been described elsewhere. Real-life-setting interventions usually require some flexibility [8] in order to fit with disparate settings. In the adaptation process therefore, the balance between fidelity to the original programme and fit with the new setting should be carefully monitored [9–11]. The most essential, effective elements of the original intervention should be maintained, but the intervention needs to fit in the new setting, i.e. the healthcare professionals’ (HCPs) working procedures [12] and organisational structure. Furthermore, in real-life settings, the intervention might be made available to a broader audience than the very restricted target group in the experimental trial [12]. Reporting the adaptation process adds to the current knowledge on making evidence-based interventions suitable for real-life setting implementation, such as implementation by HCPs from care organisations. Following this adaptation process, a feasibility and pilot phase should be conducted before performing a large-scale effectiveness evaluation [13, 14]. Information from the pilot study can be used to test the feasibility of the adapted intervention in practice and provides insight to further improve the intervention and optimise the evaluation design.

The aim of the current study is to adapt an existing efficacious experimental nutrition and exercise intervention for (frail) elderly people to a real-life setting (phase one) and to test the feasibility and potential impact of this prototype intervention in the new setting (phase two). Describing the adaptation process and pilot testing the adapted intervention will elicit valuable insights into the successful translation of efficacious interventions to real-life practice. The results of this adaptation and pilot study will be used to further refine the prototype intervention to fit the real-life practice setting and to prepare the intervention for effectiveness testing.

## Methods

The adapted intervention was designed in phase one, resulting in the prototype intervention as described in the results section. In phase two, this prototype intervention was tested for feasibility, and the potential impact of the adapted intervention in the real-life setting was assessed.

### Phase one: Design prototype intervention

In the first phase, Intervention Mapping (IM), a framework for systematic planning and adaptation of theory- and evidence-based health promotion interventions, was used to guide the adaptation to the new setting [15]. The original intervention was not systematically designed or explicitly based on behaviour change theories and behaviour change evidence. The adaptation consisted of six steps: 1) needs assessment, 2) adaptation of goals and objectives, 3) adaptation of methods and practical applications, 4) revision of programme materials, 5) planning implementation, and 6) planning evaluation [15]. In each step, we assessed whether and how the original intervention needed to be adapted to fit the new, real-life setting. Throughout adaptation, four perspectives were taken into account: relevant stakeholders, theories and supporting evidence, the implementation context, and a socioecological perspective [16]. The results of IM steps 2–5 are described in the phase one results section, IM step 6 is discussed as part of the second phase.

The adaptation process started with the involvement of all relevant stakeholders, including the intended audience (frail) older adults. As part of the needs assessment, a literature study was undertaken to obtain insight into the new implementation setting and to explore determinants of participation in exercise programmes and eating behaviour (protein intake) amongst elderly people. All available documents relating to the original intervention were studied, and a semi-structured interview was conducted with two researchers from the original intervention.

Subsequently, semi-structured focus group discussions were conducted with professionals (*n* = 5 dieticians, *n* = 3 physiotherapists) to assess whether original intervention elements would align with their standard working procedure (applicability). If they did not, the professionals were asked to provide suggestions for change. A discussion leader (EvD) and a note-taker were present during each focus group. In addition, EvD conducted semi-structured interviews with participants from the original intervention (*n* = 13) and possible future participants (n = 9), to gain insight into their experiences, needs, and desires. Interview guides for these interviews were created based on predefined intervention elements.

The results were iteratively discussed (via e-mail and face-to-face) with researchers, HCPs, and food product developers until consensus was reached about adaptations to the intervention. Researchers focused on ensuring that proposed adaptations by HCPs would not influence effectiveness. Food product developers were involved in the discussion about the nutrition programme and the selection of protein-rich products to replace standard protein drinks. The findings from these discussions relating to all IM steps are summarised in Table 1. They were used to design the adapted intervention taking the defined behaviour and behavioural determinants into account [16] and to develop intervention materials. Ethical approval for phase one was obtained from the Social Sciences Ethical Committee of Wageningen University.

### Phase two: Pilot testing the prototype intervention in practice

The pilot study was a one group pre-test post-test 12 week intervention trial among 25 elderly persons. The prototype intervention as developed in phase one was implemented during the pilot study. No sample size calculation was required as the study focused mostly on assessing the implementation process [17]. Ethical approval for the pilot study was obtained from the Medical Ethical Committee of Wageningen University. The trial was registered at clinicaltrials.gov (number NL51834.081.14). Before the start of the intervention, HCPs received implementation manuals and a short training session.

#### Participants

**Fig. 1:**
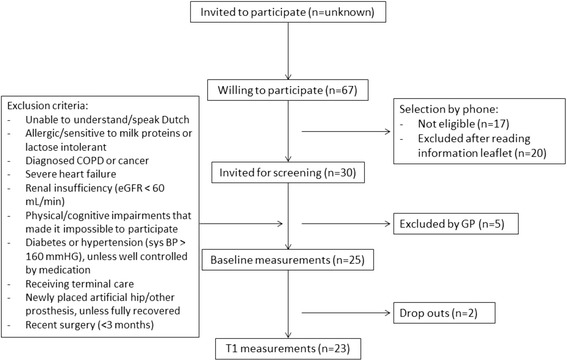
Participant flow diagram of the pilot intervention and exclusion criteria

Participants were originally recruited and provided with information via community nurses from the care organisation in the city of Harderwijk, the Netherlands. As this did not evoke enough responses for participation, a broader group of potential participants was approached and finally recruited via local organisations (e.g. choirs for the elderly) and an ad in the local newspaper. After home screening and informed consent, the participants’ general practitioner (GP) gave a final authorisation, based on the medical exclusion criteria (Fig. 1). Eligible participants were invited for the baseline measures and afterwards assigned to one of four training groups. All the community-dwelling elderly participants (aged ≥ 65 years, living in Harderwijk) were experiencing loss of muscle strength or difficulties in walking, climbing stairs, or rising from a chair.

#### Process and outcome measures

The pilot study focused primarily on process measures to study feasibility and on a number of outcome measures to assess the potential impact of the intervention in a real-life setting.

#### Process measures

The process evaluation focused on five process indicators. *Recruitment/reach* refers to the procedures to attract participants and the participants’ characteristics [18]. *Dose received* is defined as the extent to which participants were involved in intervention activities [18]. *Acceptability* indicates the extent to which the participants and the HCPs were satisfied with the intervention [19]. *Applicability* refers to the extent to which the intervention can be implemented in the real-world setting [20]. *Implementation integrity* concerns the extent to which the intervention was implemented as planned in the implementation manuals [18, 21]. Data were collected using a participant questionnaire at baseline (T0) and after 12 weeks (T1), semi-structured interviews with HCPs (T1, *n* = 2 dieticians, *n* = 4 physiotherapists, *n* = 1 coordinator within implementing organisation), and two focus group discussions with participants (T1, *n* = 6 and *n* = 8). Guides for the interviews with professionals were based on the process indicators acceptability, applicability, and integrity, and followed the content of the implementation manual. Focus group discussions with participants were less structured and assessed acceptability with regard to the exercise and nutrition intervention. EvD facilitated the focus group discussion, and a note-taker was present. To check integrity, EvD and a research assistant conducted structured observations, and the HCPs completed registration forms during the intervention. Dieticians recorded the discussed topics and advice during the consultations on registration forms. To assess the indicator dose received, physiotherapists recorded attendance and exercise intensity, and the number of repetitions during the training sessions, on registration forms. Furthermore, participants had to monitor their intake of the protein products at the designated meal occasions, using specific calendars every week during the trial.

#### Outcome measures

At baseline (T0), the Fried frailty criteria [22] and socio-demographics were assessed. Height was measured twice (to the nearest 0.1 cm) at T0 and weight was measured twice (to the nearest 0.1 kg) at T0 and T1. All other outcomes were measured once at T0 and T1. Muscle strength was tested using 3 Repetition Maximum (3RM) measures at the Leg Press and Leg Extension machines (Technogym, Rotterdam, the Netherlands), recalculated to 1 Repetition Maximum (1RM) using the assumption that 3RM weight is 90% of 1RM. At baseline, a 3RM estimation was performed to familiarize participants with the training machines and to estimate their maximal strength. At baseline also a 3RM confirmation was performed, using the estimated maximal strength of the estimation to more accurately measure to the 3RM. At T1, 3RM confirmation was performed, since 3RM weight could be estimated from their training schedules. Body composition (total lean mass, appendicular lean mass, and total fat mass) was measured using Dual-energy X-ray absorptiometry (DEXA) at Wageningen University, and bio-impedance analysis (Tanita BC-418, measuring fat free mass (FFM)), both in a non-fasting state. Physical functioning was assessed by the Short Physical Performance Battery (SPPB) [23], Timed Up-and-Go test (TUG, using the faster of two attempts) [24], and a six-minute walking test (6MWT, metres walked in six minutes) [25]. Activities of Daily Living (ADL) was measured using 22 items to assess basic (6 items), instrumental (11 items), and mobility ADL (5 items), scored on a scale of 0 (unable to perform) to 3 (able to perform without difficulty) (based on [26–30]). Quality of Life was assessed using the Short Form 36 (SF-36) [31, 32]. Summary scores for physical (physical component summary (PCS)) and mental (mental component summary (MCS)) health (scores ranging from 0–100) were calculated. All measures were performed by trained research assistants or qualified physiotherapists. Questionnaires were completed either independently by the participants or with the researchers’ assistance. Dietary intake was assessed using three-day food diaries, completed on random days. The researchers gave oral and written instructions about completing the food diaries. The T1 diaries were checked by a trained research dietician (*n* = 20 by telephone, *n* = 2 face-to-face) and coded (type of food and amount), and energy and macronutrient intakes were calculated with Compleat (food calculation programme developed by the Division of Human Nutrition; Wageningen University), using NEVO-online version 2013/4.0 [33]. The T0 diaries were coded together with the T1 diaries, using assumptions from the T1 diary check, e.g. relating to portion sizes and product brands. Overall intake and intake per meal occasion were checked.

### Data analysis

The qualitative data from the interviews and focus groups in both phases were tape recorded and transcribed verbatim. All transcripts were checked with the audio recording. Using an inductive approach [34], two researchers read the transcripts to identify important themes with regard to the intervention’s acceptability, applicability, and integrity. Coding schemes were based on those themes. Data were coded and analysed using Atlas.ti (version 7). Analysed data were used to determine whether elements of the intervention should be changed or not, and, if yes, what should be changed. These results are presented in the results section as a rationale for intervention adaptation, for both phase one and phase two.

Outcome measures were analysed using SPSS (version 22.0). Descriptives were presented as mean and standard deviation (SD), mean and 95% Confidence Interval (95% CI), or percentage. Changes in outcome measures after 12 weeks were tested by Wilcoxon signed-rank tests.

## Results

### Phase one: Intervention adaptation

Phase one resulted in the prototype intervention, consisting of a combined resistance exercise and nutrition programme for elderly participants and a training programme for the HCPs on recruitment and implementation. Table 1 presents the 5 IM steps of the adaptation process with in column one the original intervention, in columns two and three the adaptations made in phase one, and in columns 4 and 5 the adaptations made in phase two. If an element was not adapted, it can be assumed that this element was applicable and acceptable for both the participants and the HCPs.

**Table 1:** Adaptation of intervention elements for each IM step resulting from phase one and phase two

Original intervention	Phase one – design prototype intervention		Phase two – pilot test prototype intervention	
Original intervention elements	Adapt?	Adaptation to original intervention *Rationale*	Adapt?	Adaptation to prototype intervention *Rationale*
Intervention Mapping step 2: Adaptation of target population and objectives based on needs assessment (step 1)				
Target group: - age ≥ 65 years - (pre)frail - community-dwelling	Yes	Homecare-receiving clients of care organisation *Clients of care organisation where implementing HCP work.* No screening on (FRIED) frailty criteria *Simplified inclusion criteria as frailty screening is not part of regular HCP work. Assumed that care-dependent elderly are also (pre)frail.*	Yes	Broader population from the community, focus on experienced muscle weakness *Pilot had difficulty recruiting homecare clients. PTs and OR indicate better to focus on elderly who are (pre)frail or heading towards frailty; staying close to target group of original intervention.*
Specified exclusion criteria, checked by research physician	Yes	Similar exclusion criteria, but checked by participants’ own GP *Check by GP resembles real-life situation and allows large-scale implementation.*	No	
No explicit behavioural outcomes for participants	Yes	Behavioural outcomes and objectives were defined *Behavioural outcome: participants initiate and maintain participation in the exercise and nutrition intervention. As different behaviours were targeted, i.e. changing and maintaining nutrition and exercise behaviours, outcomes were specified in more detail.*	No	
Intervention Mapping step 3: Adaptation of methods and practical applications (Techniques, instruments, and methods)				
Progressive training: - work towards 75% of 1RM - check 1RM every four weeks → Method: Tailoring	Yes	Still progressive, but check 3RM and recalculate to 1RM *Implementing PTs were not confident in using 1RM in this TG; using 3RM and recalculating to 1RM is acceptable measure of strength.*	Yes	Only check 1RM at week 6 *PTs perceived 4-weekly 1RM checks as too intensive for PPs.* More focus on reaching 75% of 1RM *Training intensity in pilot not always up to 75% of 1RM.*
Trainers - encourage and motivate participants - explain purpose of exercises/nutrition → Method: Persuasive communication, arguments	No		No	
Tailored personal exercise schedule → Method: Tailoring	No		Yes	Still tailored exercise scheme, but ensure that physiotherapists train at the intensity desired in the protocol *PTs did not always use 1RM to change intensity. PTs changed lay-out of individual schedules, so it is easier to track progress.*
Monitoring protein intake using calendars → Method: Self-monitoring	Yes	Still use calendar, but now with more options to indicate consuming cheese/yoghurt/drink *DTs also perceived this as suitable and feasible way to monitor intake.*	Yes	Add more detailed monitoring, make it easier to complete calendar *Monitoring intake was not always easy for DTs due to mixed quality of completed calendars. E.g. make calendar more personally programmed, ask about compensation during meals.*
One flavour protein drink (250 mL) containing 15 g protein/drink	Yes	Range of protein-rich products (not only drinks) instead of just one drink → Method: facilitation *DTs expect that choice from a range of ordinary products would fit better with regular dietary habits and thus increase compliance. However, DTs doubt whether it is feasible to provide personalised advice over a longer period of time (maybe in the future better work with ‘standardised’ advice).*	Yes	Focus more on energy content of products *PPs experienced weight increase, so energy content of products should be taken into account in advice.* Try to incorporate more variety in products during trial *Some PPs missed product variation during trial.*
Two protein drinks a day (just after breakfast and lunch), aiming for intake of 25 g of protein per meal	Yes	DTs check during which meals protein intake should be increased and provide tailored advice on which products and portion sizes to take (in agreement with participant preferences) → Method: Tailoring *DTs and product developers emphasise the importance of tailoring protein products to individual needs and desires.*	No	
Handing out proteins for whole week drink at training, by researcher → Method: Facilitation	Yes	Protein products for whole week organised per person by DT, distributed at training by PT *Most convenient according to DT and PT, also for product storage; DT knows personal advice and PT can distribute after training session.*	Maybe	*PPs were satisfied with receiving products for the week during training. Logistics depend on whether products are provided or whether the participants should purchase them themselves.*
Arranged free transport to all trainings by volunteers → Method: Facilitation	Yes	Participants should come to training on their own *In real-life setting, more emphasis on independence. Create the training location in the community, near the participants.*	No	
Intervention Mapping step 4: Revision of programme materials (Intervention design: Delivery mode, intensity, materials)				
General				
Programme of 24 weeks	Yes	Prototype intervention of 12 weeks *Researchers saw great improvement in outcomes after 12 weeks in experimental trial. HCPs perceive this as a sufficient period to test implementation of the prototype intervention.*	Yes	Intensive intervention of at least 12 weeks, with addition of a maintenance programme *Maintenance programme was requested by HCPs and PPs, focusing on both exercise and nutrition. Some PPs indicated that 12 weeks of ‘obligations’ was long enough. PTs indicated that around 12 weeks participants reach an ‘optimum’.*
Information materials: leaflet (easy language, large font, clear information)	Yes	Adapt materials to practice setting. DTs also provide printed overview of individual advice *DTs are used to doing this with their clients, to help them remember advice.*	No	
Contact person for questions was researcher	Yes	Contact person for training was PT, for dietary intervention was DT *It is likely that these are the first persons participants will ask questions about the nutrition/exercise programme.*	Maybe	*Depends on organisational structure in implementing organisation.*
Training sessions				
Training twice a week, one hour per session	No		No	
Training supervised by researcher, assisted by trained students	Yes	Training supervised by PT, assisted by assistant PTs *(Geriatric) PTs are skilled professionals who can implement this programme in real-life. Researchers think that presence of a skilled supervisor during training sessions is important. OPs indicated that enthusiasm, social skills, and the ability to stimulate participants were important trainer qualities.*	No	
No intake consultation by trainer	Yes	Intake by PTs before start intervention *PTs perceive this as necessary to gain knowledge on possible health problems/injuries.*	No	
Training: - one trainer per two participants (individual exercise performance guidance) - same trainers all sessions	No		Yes	No 1-on-1 guidance, more flexible *According to PTs two trainers for six participants was (more than) sufficient, especially after the first few weeks. Flexible guidance was successful during pilot. PPs were satisfied with guidance. PTs’ work schedule did not allow same trainer every training session, but two different trainers was feasible.*
Training in mixed groups of maximally six elderly	No		No	
Training in gym location equipped for the trial at university	Yes	Gym location in local community, near the elderly *TG wanted training location close by. Depends on the possibilities of the care organisation; a meeting room was transformed to a gym for the intervention period as other locations were occupied.*	No	
Training session structure: - warming-up, resistance exercises, cooling-down - six training machines - no specific exercise order	No		Yes	Group-based cooling-down (stretching) *PTs added group-based stretching to enable group cohesion. According to PPs, it was a nice way to close the session.*
Researcher organised individual training schedules and trainings	Yes	Individual training schedules organised by PTs *The PTs organise the training and complete the individual training schedules during/after the training sessions. Fits their regular work.*	No	
Nutrition intervention				
Only short explanation of protein drinks at start intervention by research dietician (no real consultation)	Yes	Face-to-face consultations with DT before intervention and midway through, added (phone) consultation when needed → Method: persuasive communication, arguments *As the nutrition programme in the prototype is more extensive, DT guidance is needed to explain the need of the nutrition programme and provide advice on the protein-rich products. Individual consultations ensured two-way communication. A midway evaluation opportunity is added to evaluate and adjust the advice if necessary.*	Yes	Add contact opportunity at start intervention and include monitoring of weight and dietary compliance *DTs had to inform PPs about the protein advice again when they were handing out products. Weight gain, indicated as problem by PPs, should be monitored. PPs indicated that they sometimes compensated for the protein-rich products. Therefore, DTs should closely monitor weight and dietary compliance.*
Intervention Mapping step 5: Planning implementation				
No involvement of other organisations	Yes	Involvement of care organisation to implement intervention *Building support by discussions with organisation and involving them in adaptation process.*	No	
Recruitment by researchers, using letters to all community-dwelling elderly ≥65 years of selected cities	Yes	Recruitment by homecare nurses and care organisation’s communication department *The care organisation is also partly responsible for recruiting enough participants as it is implementing the programme.*	Yes	Provide more management support for recruiting HCPs *Pilot showed that recruitment through homecare nurses needs more attention.*
No protocol for dieticians or physiotherapists	Yes	Implementation protocol and registration forms developed for dieticians and physiotherapists *Including detailed information describing implementation of the dietary and exercise intervention. Includes detailed training protocol for PTs, although they were already familiar with exercises.*	No	
Implementing students trained by principal researcher	Yes	HCPs who recruit and implement intervention are trained by principal researcher *HCPs receive training before the intervention starts, to inform them about the implementation manual content and to train them to implement the intervention as planned. Also, the DTs and PTs meet one another during this training session, thus easing collaboration during the intervention.* Organise interdisciplinary discussion halfway through the implementation period with all implementing HCPs *HCPs indicated need to exchange experiences, so implementation could be altered if needed.*	No	
Sustainability not taken into consideration	No		Yes	Include care organisation and municipalities in project *To ensure prolonged use of intervention, after (cost)- effectiveness is shown.*

*HCPs* healthcare professionals, *PTs* physiotherapists, *OR* original intervention researchers, *GP* general practitioner, *1RM* 1 repetition maximum strength, *TG* target population of the intervention in real-life setting, *3RM* 3 repetition maximum strength, *PPs* Pilot study participants, *DTs* dieticians, *OPs* original intervention participants

There were four major changes to the original intervention in the prototype intervention. First, behaviour change goals for both participants and HCPs were specified, as these were not explicitly specified in the original intervention. Second, theoretical methods (general techniques for influencing change in determinants of behaviour [16]) were identified to provide a theoretical foundation for the intervention’s activities. These included tailoring (matching the intervention to participant characteristics [35]), persuasive communication (using arguments to guide an individual towards adoption of an action [36]), and facilitation (creating an environment that reduces barriers to action [37]). Third, an extensive nutrition programme with a dietician replaced the protein supplementation in the original intervention. In order to fit better with the participants’ regular dietary pattern, protein intake was to be increased using a range of protein-rich products, instead of just one drink. Guidance by a dietician was perceived necessary to enable the participants to perform this nutrition intervention. Finally, a training programme for implementing professionals was designed to ensure quality implementation. As the original intervention had not been implemented in practice, the training programme for HCPs was an element of the adapted intervention.

Several elements were already applicable to the practice setting or were important to retain according to, e.g., the researchers. For example, HCPs were already used to encouraging and motivating elderly people, and this was also perceived as important by the original intervention’s participants. Physiotherapists and researchers agreed that progressive training and two training sessions of one hour a week were important to achieve results and that in a frail elderly population intensive guidance and a small training group were important. Physiotherapists and researchers deemed it necessary to use a tailored training schedule based on 1RM and individual possibilities. The original study participants perceived the group-based training and being informed about improvements in strength during four-weekly 1RM checks as motivating. Furthermore, monitoring intake of protein products was seen as important, and dieticians perceived calendars to be the easiest way to monitor this.

The adapted prototype intervention consisted of the following parts:

- *Resistance exercise intervention:* The participants performed progressive resistance exercise twice a week (one day’s rest in between) for one hour, guided by physiotherapists. Training groups consisted of 5–7 participants. Each training session included warming-up (5 min easy biking on a home trainer, 60 rpm), six strength exercises (leg press, leg extension, lat pulldown, vertical row, chest press, and pec dec), and cooling-down (5 min easy biking on a home trainer, 60 rpm), similar to the exercise protocol in the original study. Training schedules were based on personal maximum strength tests. According to the protocol, the leg exercises were performed with 4 sets of 8–12 repetitions, and physiotherapists should increase the intensity from 50 to 75% of 1RM. The other exercises were also performed in 4 sets with 8–12 repetitions, but in a less progressive manner.
- *Nutrition intervention:* The nutrition programme included two consultations with a dietician (at the beginning and halfway through), and an additional consultation if needed. Dieticians formulated a personally tailored nutrition intervention with protein-rich dairy products for breakfast and lunch (the second bread-meal), aiming to achieve an intake of 25 g of protein to evoke the most optimal muscle protein synthesis response in these main meals. Participants received the recommended protein products, such as cheese, dairy drinks, and Greek yoghurt, for free during the study. These products were either supplements to their meals or substitutes for other products.
- *Training for recruiting professionals:* The care organisation’s homecare nurses were instructed about recruitment at a training session of approximately 30 min and given an information leaflet explaining the intervention and their recruitment tasks. The nurses invited care-receiving elderly persons to participate in the intervention. The progress of the recruitment phase was monitored, and nurses received a recruitment reminder via e-mail.
- *Training for implementing professionals:* Before the intervention started, the participating physiotherapists and dieticians received their implementation manuals and a training session of 1.5 h to instruct them on the intervention and implementation. Halfway through the intervention, both professional groups compared their experiences with implementing the programme in an interdisciplinary discussion on problems and solutions.

### Phase two: Pilot study

Phase two described the evaluation results of the tested prototype intervention, including the resulting adaptations to the intervention as presented in columns four and five of Table 1.

#### Reach/recruitment

In total, 67 persons indicated interest in participating in the intervention (eight through homecare nurses, 59 through other recruitment means). After screening by the researcher and a check for exclusion criteria by their GP, 25 participants were eligible to participate and started with the baseline measures (Fig. 1). Non-eligible participants (*n* = 42) did not differ from included participants, with a mean age of 73.5 ± 7.4 years (*n* = 36) and 40.5% males. After three weeks, two participants dropped out, due to health issues and time constraints. All remaining 23 participants completed the measures after the intervention (T1).

Eligible participants were on average 74 years old, and 36% were male (Table 2). Eleven participants were non-frail, twelve were pre-frail, and two were frail based on the Fried frailty criteria [22]. All participants were of Dutch ethnicity, and half lived with a partner. At baseline, participants were very motivated to participate in the intervention (score of 4.6 on a scale of 1–5). Fifteen participants did not receive care, and the others received mostly domestic help and/or informal care.

**Table 2:** Baseline characteristics of participants (*N* = 25) of the pilot intervention

Characteristic	Mean ± SD or N (%)
Age	74.1 ± 6.8
Gender: Male	9 (36)
Frailty status	
– Non-frail	11 (44)
– Pre-frail	12 (48)
– Frail	2 (8)
Education level^a^	
– Low	10 (40)
– Intermediate	14 (56)
– High	1 (4)
Ethnicity: Native Dutch	25 (100)
Marital status: Married/living together	13 (52)
Motivation at baseline^b^	4.6 ± 0.7
Alcohol: Drinker (≥1 day/week)	14 (56)
Smoking: Current smoker^c^	2 (8)
One or more morbidities^d^	23 (92)

^a^Based on the highest level of education completed, divided into three categories: low (primary school or less), intermediate (lower/medium vocational education, high school), and high (higher vocational education, university)

^b^Scale 1 (totally unmotivated)–5 (very motivated)

^c^Current smoker or stopped smoking < 1 year ago

^d^Diagnosed by GP in last 12 months. Main morbidities are: high blood pressure (*n* = 11), joint pain (*n* = 8), visual impairments (*n* = 8), and back problems (*n* = 6)

#### Acceptability and dose received

The intervention received high acceptability ratings from both the participants and professionals (8.7 and 7.6, respectively). Focus group discussions and the T1 questionnaire showed that participants were pleased with both the exercise and the nutrition programme. They described the professionals’ guidance, the (group) ambiance during training sessions, tailoring of the exercise programme, being informed about strength increases, and the characteristics of the supplementary products as positive points. However, small points for improvements related to short intervals between the two training days, some inappropriate training machines for their age group, lack of variation in protein products, and too high consumption amounts of the products, as well as undesired weight gain. They expected that maintaining a protein-rich diet after the project would be quite easy, and they would use similar products to the ones received during the intervention. With regard to continuing to exercise, participants indicated that they wanted to do so in small groups with likeminded older adults, with supervision, and without very high costs.

Professionals were very positive about, among other things, the combination of exercise and nutrition, and the participants’ enthusiasm, but indicated some factors for improvement, as shown in Table 1. They perceived the interdisciplinary group discussion as a very useful way to compare experiences and elicit points for attention in the last six weeks of the intervention.

Participants attended on average 86.4% of training sessions, and all participants received both an intake (mean duration 30 min, *n* = 20) and a midway evaluation consultation (mean duration 16 min, around week 6, *n* = 21) with the dietician. According to the registration forms, only one participant received an additional consultation. The dietician adjusted the advice for 10 participants (43.5%) during the evaluation consultation, mainly because of suspected weight gain. Intensity of the leg exercises was on average 62% of 1RM, in three sets. Self-reported data from participants showed that they consumed the recommended products during on average 94% of meals (Table 3).

**Table 3:** Participants’ and professionals acceptability of the pilot intervention and dose received by participants

	Participants (mean ± SD)	Professionals (mean ± SD)
Overall intervention	*n* = 23	*n* = 7
Acceptability	8.7 ± 0.7	7.6 ± 0.6
Because of my participation in this project…		
I received a lot of individual attention (1–5)^a^	4.2 ± 0.8	
I feel stronger (1–5)^a^	3.7 ± 1.1	
I feel better physically (1–5)^a^	3.7 ± 0.9	
I feel better mentally (1–5)^a^	3.6 ± 1.0	
Exercise programme		*n* = 4
Acceptability	8.9 ± 0.8	7.5 ± 0.4
Because of my participation in this project…		
I enjoyed exercising (1–5)^a^	4.5 ± 0.7	
I could exercise with a goal (1–5)^a^	4.3 ± 0.7	
How satisfied were you with…		
the fact that the exercises were in a training group? (1–5)^b^	5.0 ± 0.2	
the duration of the training sessions (1 h)? (1–5)^b^	4.9 ± 0.3	
the supervision during the training sessions? (1–5)^b^	4.9 ± 0.5	
the exercises you had to perform? (1–5)^b^	4.8 ± 0.4	
the division of the training sessions over the week? (1–5)^b^	4.1 ± 1.1	
Nutrition programme		*n* = 2
Acceptability	8.4 ± 1.0	7.5 ± 0.7
How satisfied were you with…		
the extent to which the dietician took your dietary preferences into account? (1–5)^b^	4.6 ± 0.8	
the possibility to adjust the advice? (1–5)^b,c^	4.7 ± 0.7	
the intake consultation with the dietician? (1–5)^b^	4.5 ± 0.9	
the midway evaluation consultation? (1–5)^b,c^	4.6 ± 0.8	
the products the dietician recommended? (1–5)^b^	4.5 ± 0.8	
	Dose received	
Exercise programme		
Training attendance (# of sessions, (% of total))	19.9 (86.4%)	
Exercise intensity (% of 1RM) – Leg Press (mean ± SD)	61.4 ± 6.4	
Exercise intensity (% of 1RM) – Leg Extension (mean ± SD)	62.4 ± 12.4	
Nutrition programme		
Participants receiving intake (n (%))	23 (100%)	
Participants receiving evaluation consultation (n (%))	23 (100%)	
Compliance with taking products (mean ± SD)^d^	94.2 ± 8.1	

^a^Score 1 (totally disagree) to 5 (totally agree)

^b^Score 1 (very dissatisfied) to 5 (very satisfied)

^c^ *n =* 22

^d^Percentage of meals during which recommended products were consumed; based on an average of 53 days of completed calendars

#### Integrity

A broader recruitment strategy than initially planned was used, because not enough participants were recruited among homecare clients. Overall, HCPs implemented the programme as planned in the implementation manuals, although Table 1 shows some adjustments to the protocol during the pilot. The physiotherapists did not engage in real intake consultations, as researchers provided them with the relevant background information about participants. The 1-on-1 guidance was omitted in the first weeks of the intervention, because having less structured group guidance was a better fit with their usual way of working and most participants did not need such structured guidance as they were quite fit and independent. Participants indicated that they appreciated the guidance received during the training sessions, and therefore this adaptation to the manual was not seen as a problem. The newly added group-based cooling-down was perceived as pleasant by the participants, as it was also a moment of interaction and laughter. Physiotherapists indicated that they motivated and encouraged participants, provided positive feedback during the training sessions, and showed the 1RM progression to the participants; this was also welcomed and confirmed by the participants.

Some participants had an intake consultation with the dietician in the first week of the intervention instead of before the programme started. Also, it appeared that the intake consultation alone did not provide the participants with enough information on how and when to take the products, so extra contact was needed at the start to repeat the explanation of the nutrition intervention. Moreover, participants asked the dieticians small questions if the latter happened to be around during training sessions. Furthermore, participants’ complaints triggered the dieticians to pay specific attention to weight gain, and they monitored participants’ weight during the follow-up consultation. Also, some participants indicated that they missed variety in the products provided and sometimes skipped foods from their ‘regular’ diet, so variation in products and close monitoring of weight and dietary compliance is a point for attention in the future.

#### Applicability

All professionals perceived the intervention as matching their professional skills and knowledge and were willing to continue working with this programme. However, as can be seen in Table 1, there were some points for improvement, such as adaptations to the format of individual training schedules and training machines that are more suited to an elderly population. Also, the HCPs emphasised specific skills that HCPs should have when implementing this intervention: good communication skills, familiarity with the participants and their comorbidities/level of ability, and ability to motivate participants. For the training sessions, physiological knowledge is needed to prevent injuries.

#### Outcome measures

Participants increased significantly in leg strength during the intervention, with an average increase of 24.9% in leg press strength and 39.1% in leg extension strength. No change in total lean mass and a non-significant decrease in appendicular lean mass were observed, whereas weight and total fat mass increased significantly. Participants showed significant improvements in all three physical functioning tests. There were no significant changes in basic, instrumental, mobility (data not shown) or total ADL. Participants showed a slight, non-significant increase in the PCS of the SF-36. Results from the three-day food diaries showed that participants significantly increased their daily protein intake to 1.2 g of protein/kg-bodyweight/day. Protein intake increased significantly during breakfast and lunch to 24.1 and 29.9 g, respectively. On the days the diaries were completed, the desired intake of 25 g of protein per meal (breakfast and lunch) was achieved by, respectively, 45.5 and 86.4% of participants (compared to, respectively, 13.6 and 22.7% at baseline) (Table 4).

**Table 4:** Twelve-week changes in health outcomes and dietary intake of the pilot intervention participants

	N	Baseline mean (95% CI)	ΔT1-T0^a ^mean (95% CI)	*p*-value^b^
Strength^c^				
– 1RM Leg press (kg)	23	137.4 (120.8–154.0)	31.7 (20.6–42.8)	0.000
– 1RM Leg extension (kg)	22	52.6 (44.2–60.9)	17.8 (13.6–22.0)	0.000
Anthropometrics				
– Weight (kg)	23	85.1 (79.0–91.3)	0.9 (0.2–1.5)	0.007
– Total lean mass (kg)	23	48.8 (44.7–53.0)	–0.1 (–0.6–0.3)	0.447
– Appendicular lean mass (kg)	23	21.4 (19.3–23.4)	–0.3 (–0.6–0.0)	0.073
– FFM (kg)	20	52.4 (47.4–57.4)	0.4 (–0.7–1.4)	0.513
– Body mass index	23	29.4 (28.0–30.9)	0.3 (0.1–0.5)	0.009
– Total fat mass (kg)	23	33.2 (29.5–36.9)	0.7 (0.1–1.4)	0.029
SPPB				
– Total score	23	9.1 (8.3–9.9)	0.7 (0.0–1.3)	0.047
– 4 m walk (sec)	23	4.1 (3.8–4.4)	0.1 (–0.4–0.5)	0.831
– Repeated chair rise (sec)	18	17.6 (15.2–19.9)	–3.6 (–5.8-–1.4)	0.002
TUG (sec)	23	10.6 (9.1–12.2)	–1.3 (–1.8-–0.8)	0.000
6MWT (m)	23	384.5 (357.9–411.1)	27.5 (12.8–42.3)	0.002
ADL (total score)^d^	23	2.8 (2.7–2.9)	0.0 (–0.1 – 0.0)	0.407
Quality of life^e^				
– MCS	23	57.2 (54.0–60.5)	0.4 (–2.9–3.6)	0.879
– PCS	23	42.9 (38.5–47.3)	2.7 (–0.3–5.8)	0.073
Dietary intake				
– Energy (MJ)	21	7.6 (6.6–8.7)	0.7 (–0.1–1.5)	0.106
– Protein (g)	21	79.9 (67.6–92.3)	23.1 (10.2–36.0)	0.003
– Protein (g/kg-bw/day)	21	0.96 (0.81–1.12)	0.29 (0.13–0.45)	0.002
– Protein breakfast (g)	21	15.1 (11.5–18.6)	9.0 (3.9–14.0)	0.003
– Protein lunch (g)^f^	21	19.4 (15.8–23.1)	10.5 (6.2–14.7)	0.000
– Protein dinner (g)	21	37.1 (31.7–42.5)	–0.7 (–5.0–3.7)	0.986
– Protein (en%)	21	17.8 (15.5–20.2)	3.5 (1.6–5.4)	0.002
– Fat (en%)	21	31.2 (28.2–34.2)	0.6 (–3.5–4.8)	0.715
– Carbohydrates (en%)	21	46.0 (41.9–50.0)	–5.9 (–10.1-–1.7)	0.004

^a^Change between baseline (T0) and follow up (T1)

^b^Wilcoxon signed-rank test

^c^Baseline score is 1RM estimation, as 1RM confirmation was not documented

^d^Mean of score of basic, instrumental, and mobility ADL, score range 0 (cannot do)–3 (can do completely independently)

^e^MCS is Mental Component Summary, PCS is Physical Component Summary

^f^Lunch is second bread-meal

## Discussion

This adaptation and pilot study showed that a highly structured experimental intervention can be successfully adapted for implementation in a real-life setting. Adaptations to the experimental intervention related mostly to the design of training for implementing and recruiting professionals, design of a dietician-guided nutrition programme, and organisation of the training sessions. The prototype intervention was perceived as highly acceptable by both participants and professionals, and applicable to implement in Dutch healthcare practice. Furthermore, the findings of the pilot study showed indications of positive impact on muscle strength and physical performance, but not on muscle mass, in older adults. The pilot study also provided insight into intervention elements that may need further adaptation, such as the recruitment strategy and parts of the HCP training to implement the intervention.

Adapting evidence-based health promotion interventions can be a challenge, especially if the interventions are not systematically described [13] and not based on social psychological theories, and if evaluation studies measuring their efficacy do not take into account both internal and external validity [9]. Efficacy trials that use a very strict protocol and are delivered in research settings by research staff are not directly appropriate for implementation in practice [9]. Successful adaptation requires insight into the ideas and implementation experiences of the designers of the original intervention, as well as support from the intended implementers of the intervention. A systematic adaptation approach provides insight into effective intervention elements by establishing which parts of the intervention have to remain, and which elements need adaptation to fit the new setting.

Although the prototype intervention was mostly feasible to implement as planned, the pilot study evaluation elicited some adaptations to improve fit to the practice setting and HCP working procedures. As the nutrition intervention was added to the prototype intervention, the pilot provided valuable suggestions to improve feasibility. Points for attention in subsequent intervention implementation are monitoring body weight, adding sufficient product variety, monitoring compliance, and providing ample guidance at the start of the programme. The physiotherapists adapted implementation of the training sessions to allow more flexibility and leave room for social interactions in the group. It is expected that, with the adaptations in phase two, the prototype intervention is ready to be tested on effectiveness in practice. However, when the intervention is implemented by other organisations, it is expected that commitment to properly adopt and implement the intervention will have to be created among the organisation’s professionals [8] and that the implementation manuals will have to be fine-tuned to the specific organisation on aspects such as organisational structure and HCP task divisions.

Recruitment for the pilot study required some effort, as often reported in studies in elderly populations [38]. Relying on homecare nurses to recruit the specific group of homecare-receiving elderly did not go as intended, and the fact that participants had to travel somewhere to attend the group training sessions might have led to recruitment of a broader, possibly fitter, group of participants than when only homecare recipients were included. This pilot showed that both non-frail and pre-frail individuals experienced benefit from the intervention with regard to physical functioning and strength (data not shown). As stated by Glasgow et al. [8], practice trials should work in, and appeal to, a broad target audience. Although all participants were positive about the intervention, recruitment of non-care-receiving elderly was easier, and professionals indicated that it would require more effort to implement the intervention if only (pre)frail individuals were involved. Selecting the ideal target group for this intervention means finding a balance between elderly persons who are very willing to participate and those for whom the original intervention was developed but are more difficult to reach. Given these issues, the proposed effectiveness trial will focus on the original intervention target group, (pre-)frail elderly. In order to facilitate recruitment, the pilot participants’ positive experiences might be included in the recruitment materials to make the programme more attractive. Also, extra attention will be given to training homecare nurses about recruitment.

The pilot study showed indications of positive effects on several outcome measures. Strength and physical functioning improved, as also found in both groups in the original intervention [7]; this accords with Cermak et al.’s meta-analysis of protein supplementation and resistance-type exercise training [5]. The changes in leg strength results are comparable to findings reported in Peterson et al.’s meta-analysis of the effect of resistance exercise on muscular strength in older adults [39], with a 29 and 33% increase in leg press and knee extension strength, respectively. Compliance was high for both the nutrition and the exercise intervention in the pilot, although training intensity (62% of 1RM and only three sets instead of four) was slightly lower than in the original. Even though the pilot study participants were not all (pre-)frail and the intervention was implemented in a more flexible way, our results give indications of retained effectiveness in practice. The pilot did not detect changes in lean body mass in the participants, whereas it is assumed that exercise [40] and sufficient protein intake [41] increase muscle protein synthesis and muscle mass accretion in elderly people. A possible explanation for not finding an increase in muscle mass might be the protein intake, which was still low for breakfast. The original study resulted in a slightly higher protein intake than the pilot study [7]. Previous studies suggest that 25–30 g protein per main meal is needed to maximally stimulate muscle protein synthesis and increase muscle mass in older adults [42]. In addition, DEXA measurements were performed in a non-fasting state, with no standardisation of meals or drinks, and not at the same time of day at both time points. This may have influenced the accuracy of the measures. These aspects should be taken into consideration in the proposed effectiveness study.

Even though the current study gave indications of potential impact of this adapted intervention in a real-life setting, further research is needed to test the (cost)-effectiveness of the up-scaled intervention in practice. As implementation can differ between settings, it is important to test the effectiveness of the adapted intervention in multiple locations and within multiple organisations. Maintenance of intervention implementation should be ensured on both the professional and the organisational level [12]. Establishing a broad network of stakeholders is important to facilitate future continuation of the project. This network should include implementing care organisations, the target group, and possible other stakeholders in the field of nutrition and/or exercise. Funding is another important issue to be addressed by the involved stakeholders to ensure further implementation of the intervention in the local setting. Pilot participants indicated that they would like to continue, so ideally a follow-up programme should be developed. This should facilitate a fluent transfer to regular exercise and nutrition guidance in order to help participants to sustain their newly adopted healthy lifestyle after the intervention. Additional research is needed to assess specific participant needs in maintaining this lifestyle.

### Strengths and limitations

Although the size of the current study was not intended to provide sufficient power to detect differences in outcomes, and no control group was included, most of the outcome measures showed significant, positive effects. This indicates that translation of the intervention was successful and that effective elements of the intervention were retained. Furthermore, as the evaluation showed that in general the intervention was applicable and acceptable for the professionals and participants, it seems that a good balance was achieved between integrity to the original intervention and fit with the new setting. Limitations of the pilot include testing feasibility in only one location and not fully reaching the desired target population of homecare-receiving elderly. Nevertheless, the pilot provided essential insights into aspects to consider in recruiting (pre-)frail elderly, and motivating the target population remains a point for attention in the proposed effectiveness study.

## Conclusion

The clinical intervention was successfully adapted from a research setting to a real-life setting in Dutch primary healthcare using a concise Intervention Mapping approach, and perceived implementation feasibility was tested in a pilot study. Proposed adaptations to the prototype intervention after the pilot study relate mainly to guidance by physiotherapists and dieticians. The study showed potential impact on muscle strength and physical functioning outcomes, indicating effectiveness after adaptation. As the results from the pilot study are promising, in the next phase the adapted intervention will be tested for (cost) effectiveness in a larger, multicentre randomised controlled trial.

## Abbreviations

1RM: 1 repetition maximum
3RM: 3 repetition maximum
6MWT: Six minute walking test
95% CI: 95% Confidence interval
ADL: Activities of daily living
BMI: Body mass index.
DEXA: Dual-energy X-ray absorptiometry
DTs: Dieticians
FFM: Fat free mass
GP: General practitioner
HCPs: Healthcare professionals
IM: Intervention Mapping
MCS: Mental component summary
NEVO: Dutch nutrient composition table
OPs: Original intervention participants
OR: Original intervention researchers
PCS: Physical component summary
PPs: Pilot study participants
PTs: Physiotherapists
SD: Standard deviation
SF-36: Short Form 36
SPPB: Short Physical Performance Battery
TG: Target population of the intervention in real-life setting
TUG: Timed Up-and-Go test
